# From communication to action: using ordered network analysis to model team performance in clinical simulation

**DOI:** 10.1186/s12909-025-07062-5

**Published:** 2025-04-03

**Authors:** Vitaliy Popov, Lauryn R. Rochlen

**Affiliations:** 1 Department of Learning Health Sciences, University of Michigan Medical School, University of Michigan, School of Information, Victor Vaughan, 217, 1111 Catherine St, Ann Arbor, MI 48109 USA; 2 School of Information, Ann Arbor, MI USA; 3 Department of Anesthesiology, University of Michigan Medical School, Ann Arbor, MI USA

**Keywords:** Team communication, Ordered network analysis, Clinical Simulation, Anesthesiology Acute Care Teams

## Abstract

**Background:**

Effective team communication is crucial for managing medical emergencies like malignant hyperthermia (MH), but current assessment methods fail to capture the dynamic and temporal nature of teamwork processes. The lack of reliable measures to inform feedback to teams is likely limiting the overall effectiveness of simulation training. This study demonstrates the application of ordered network analysis (ONA) to model communication sequences during the simulated MH scenario.

**Methods:**

Twenty-two anesthesiologists participated in video-recorded MH simulations. Each scenario involved one participant as the primary anesthesiologist with confederates in supporting roles. Team communication was coded using the Team Reflection Behavioral Observation (TuRBO) framework, capturing behaviors related to information gathering, evaluation, planning, and implementation. ONA modeled the sequences of these coded behaviors as dynamic networks. Teams were classified as high- or low-performing based on timely dantrolene administration and appropriate MH treatment actions. Network visualizations and statistical tests compared communication patterns between groups.

**Results:**

Five of 22 teams (23%) were high-performing. ONA revealed high-performers transitioned more effectively from situation assessment (information seeking/evaluation) to planning and implementation, while low-performers cycled between assessment behaviors without progressing (*p* = 0.04, Cohen’s d = 1.72). High-performers demonstrated stronger associations between invited input, explicitly assessing the situation, stating plans, and implementation.

**Conclusions:**

Integrating video coding with ONA provides an innovative approach for examining team behaviors. Leveraging ONA can uncover patterns in communication timing and sequences, guiding targeted interventions to improve team coordination in various real-world clinical and simulated settings (e.g., operating room, EMS, ICU).

## Background

Reliable analysis of team processes and communication patterns both clinical and simulation-based team training settings is crucial for understanding the dynamic nature of teamwork and informing educational interventions. The current standard assessment practices in both clinical and simulation-based team training settings are based on global rating scales and the third-party observation of specific verbal and nonverbal behaviors, assessed as static constructs [1]. The observation tools generally consist of two main approaches: behavioral marker systems and coding schemes [2–4]. These are labor-intensive, obtrusive, and prone to personal judgment and error. This limitation results in healthcare professionals receiving feedback that is of variable quality—often generalized, inconsistent, and highly dependent on human observers or simulation instructors [5, 6].

Most importantly, the current approaches fail to capture team processes, which are innately dynamic, interdependent, and temporal [7–9]. For example, crisis checklists evaluate if steps were completed but not how and in what order the steps were carried out. With feedback being essential for learning and development, this limits the effectiveness of simulation training. Quantifiable markers of teamwork and communications are necessary to develop focused educational interventions and coaching strategies [10].

Perioperative emergencies, such as malignant hyperthermia (MH), provide a suitable context to study team communication patterns. MH is a rare complication of general anesthesia that could develop in any patient, and many care providers may have limited opportunities for early recognition, treatment, and management due to minimal clinical experience with MH [11]. While technical skills are crucial when facing emergencies like MH, nontechnical skills (including communication, leadership, and situational awareness) are equally important for improving patient outcomes. Simulation-based instruction has been generally accepted as playing an important role in MH training. Multiple empirical studies found that simulation-based learning improved knowledge, confidence, and skills in early recognition, treatment, and management of MH [12–14].

To bridge this knowledge gap in understanding dynamic team communication, one promising approach is to analyze the patterns of speech acts performed by team members during simulated medical emergencies. Speech acts refer to utterances that serve a function or purpose in communication, such as requesting information, providing an evaluation, or stating a plan [15, 16]. Capturing and analyzing speech acts allows researchers to map the flow of verbal communication within a team. A few previous studies have coded speech acts in simulation studies, such as trauma communication [17], surgery [18] and oncology settings [19]. In a similar vein, a new structured observation framework called TuRBO (Team Reflection Behavioral Observation) was recently developed through a rigorous theoretical and empirical approach [20]. TuRBO delineates key speech acts pertinent to acute care teams, categorizing utterances as seeking information, evaluating information, stating a plan, expressing concern, providing reassurance, and resolving conflict. This allows raters to efficiently tag verbal communications during or after simulations.

The connections between TuRBO-coded speech acts can then be analyzed using an innovative methodology called Ordered Network Analysis (ONA). ONA models sequences of coded behaviors as networks, quantitatively identifying the frequency of speech acts, order, and temporal connections between them [21, 22]. Specifically, ONA takes coded data as input, identifies and measures connections among coded items, and visualizes the structure of connections in a metric space that enables both statistical and visual comparison of networks [23].In contrast to conventional coding approaches that treat behaviors as isolated variables, ONA provides a dynamic map that captures the interdependent nature of team communication [21, 22, 24]. By applying ONA to link TuRBO-coded speech acts, researchers can uncover patterns in the timing and co-occurrence of key communications which may guide targeted interventions to improve coordination and communication. This network modeling enables detailed comparisons of the communication workflows between different teams (e.g., higher- and lower-performing; variation in team composition, scenario difficulty, etc.). Educators and researchers can also trace how specific behavior patterns correlate with skill acquisition as teams participate in multiple simulations over time.

This study was guided by the following research question: *How can ordered network analysis be applied to quantify and visualize communication patterns during a simulated anesthesia crisis management scenario?* The primary outcome measured is team performance, defined as administering dantrolene within 10 min from the start of the scenario and performing other appropriate treatment actions for MH. The variables examined to understand this outcome are the specific speech acts (e.g., information seeking, evaluation, planning, and implementation) used by the team members and the sequential patterns in which these speech acts co-occurred during the simulated crisis.

## Methods

### Participants and setting

This study examined team interactions during simulated crisis scenarios involving a total of 22 anesthesiologists (15(68%) males and 7(32%) females) alongside confederates who played standardized roles. The simulation teams consisted of one board-certified anesthesiologist as the primary provider being evaluated, with other participants serving as confederates in roles such as surgeon and secondary anesthesiologist. As part of the Maintenance of Certification in Anesthesiology (MOCA©), anesthesiologists who were board certified after 2000 were required to participate in a simulation course at a simulation center endorsed by the American Society of Anesthesiologists (ASA). All anesthesiologist participants were board certified and attended a simulation course at a midwestern academic medical center over a 5-year period. Date of initial certification was obtained from the American Board of Anesthesiologists (ABA) Physician Directory. The study was approved by the Institutional Review Board (HUM00194473). The need for consent to participate in this study was waived by the IRB as it involved the secondary use of video data previously collected with participant consent for education and research purposes. The simulations were conducted on a high-fidelity mannequin in the Operating Room training space at the Clinical Simulation Center at the University of Michigan. Each scenario was overseen by the course director, who guided the confederates and controlled the mannequin. Each scenario was tailored to last 15–20 min.

Our sample size was determined by the total number of simulation sessions conducted over a five-year period at our institution. Each simulation session contained on average 315 lines of dialogue which provided sufficient conversational data to model with the 8 TuRBO codes and build a reliable matrix for ONA, which could help identify statistically meaningful patterns in the interaction networks [25].

### Learning scenario

Since we used MOCA simulation course data, the learning scenario was structured to primarily evaluate individual anesthesiologist performance. The scenario involved the intraoperative management of a 36-year-old female who was diagnosed with acute cholangitis, and is now undergoing laparoscopic cholecystectomy. The primary anesthesiologist role was assigned to one course participant being evaluated. The surgeon and secondary anesthesiologist (assistant) were played by other course participants. The role of surgeon served as a confederate along with the course instructors. Confederate roles were instructed to maintain a relatively passive role, providing input only when directly addressed or at predetermined trigger points in the scenario. This standardized approach was chosen to maintain consistency across evaluations and provides an ideal testing environment for applying our analytical framework. The scenario begins with the primary anesthesiologist taking over the case from one of the course instructors. The patient is receiving general anesthesia and the procedure has already begun. The procedure is complicated by surgical difficulties resulting in the surgeon requesting additional muscle relaxant and increased insufflation pressures. There is also concern that the patient is developing sepsis given the significant gallbladder infection.

The patient develops malignant hyperthermia (MH) as the simulated scenario progresses. The primary anesthesiologist must recognize this and begin appropriate treatment. Treatment algorithms for MH are well known and broadly available [26, 27]. Definitive treatment includes stopping the triggering agents, administering dantrolene, and supportive care.

### Data analysis

#### Team performance measurement

As these simulations were conducted for individual certification purposes, performance metrics were focused on the primary anesthesiologist’s crisis management skills. All recorded scenarios were reviewed for specific care team actions. These actions include: Time to call for help, Time to ask surgeon to pause operating, and Time to administration of first dose of dantrolene. These measures were selected for analysis as they are the most critical to appropriate treatment of MH. It was also noted if the anesthesia team required a prompt from surgeon to achieve the correct diagnosis of MH. The timer started after the handover to the primary anesthesiologist was completed. Teams were categorized into high-performing and low-performing based on the time taken to administer the first dose of dantrolene, and the expert observations of a board-certified anesthesiologist (LRR). The following key criteria were used when reviewing video recordings of each team: timely diagnosis, the use of cognitive aids, utilization of additional help, communication effectiveness, and resource use. For example, teams that performed well demonstrated clear communication by asking surgeons to pause and conveying concerns, enabling timely interventions. They diagnosed MH by attending to symptoms like increased temperature and CO2 levels and efficiently utilized resources such as the MH cart and hotline. In contrast, missed actions included communication deficiencies where leaders failed to prompt a pause or convey concerns adequately, resulting in delays. Many teams also postponed diagnosing MH despite clear symptoms and did not use available resources promptly, further delaying appropriate treatment.

### Ordered network analysis

We used a three-step process to perform the ONA: (1) data transcription and segmentation, (2) directed content analysis, and (3) network analysis.

#### Step 1: data transcription and segmentation

**Fig. 1:**
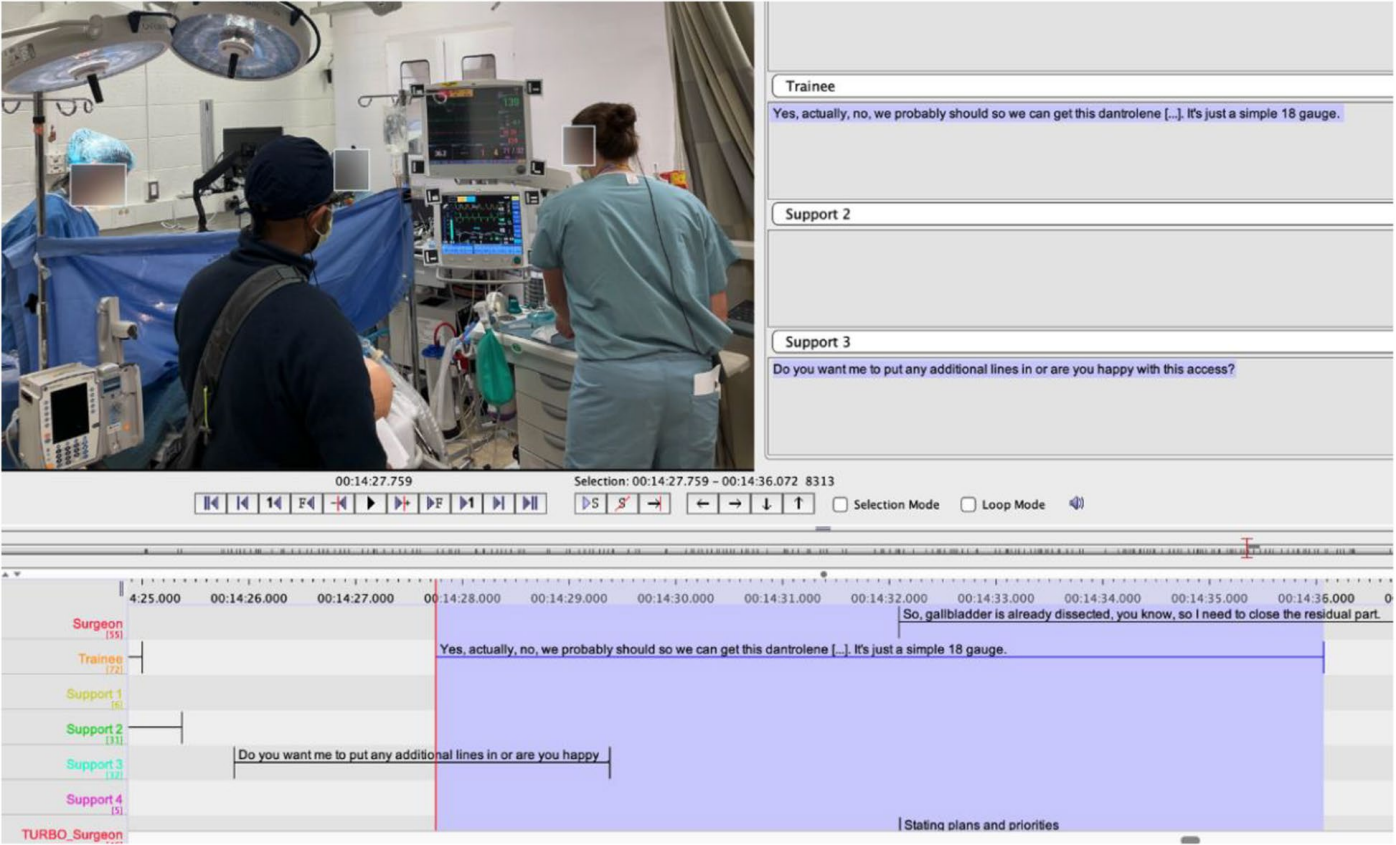
A screenshot of the ELAN software with one segment depicting surgeon, lead anesthesiologist and support team member

As an initial step of data preparation for ONA, we transcribed and segmented the videos to identify portions of the discourse of each team during the simulated scenario. These segments were done at the sentence level as the meaningful unit of analysis with the use of ELAN software (Fig. 1) [28, 29].

#### Step 2: directed content analysis

The segmented data were then annotated using the Team Reflection Behavioral Observation (TuRBO) System for acute care teams (see Table 1). The TuRBO is a framework for systematically observing and measuring in-action team reflection, defined as the process of team members briefly stepping back from tasks during a dynamic event to collectively gather information, assess the situation, and do short-term planning. Developed through an iterative process of literature review, video observation, and reliability testing, TuRBO captures team reflection through three dimensions of seeking information, evaluating information, and planning. Initial validity evidence shows TuRBO provides meaningful assessment of teamwork tied to performance, with descriptive behavioral markers that can translate to training acute care teams. The authors (VP, LRR) worked through each TuRBO category to identify which statements made by the anesthesia teams would be appropriate. An implementation category was added, given the importance of recognition of MH and then also acting to implement appropriate treatment. These categories were not shared with participants.

**Table 1:** Team Reflection Behavioral Observation (TuRBO) system adapted from Schmutz et al. [20] with direct quote examples from the study

	TuRBO code	Description	Example from the study
Seeking Information	Actively inviting input	All statements that request information from the team about the current event and invite team members to provide information and share ideas	“What type of abx did this patient get? Do we need a repeat dose? Can you add abx? (Trainee; Video MH 9–11)
	Expressing uncertainty	Expressions of uncertainty with an implicit invitation to share information	“Is there anything we are missing? Is there anything else we should be doing” (Trainee; Video MH 10-R)
Evaluating Information	Stating a working hypothesis	Clear formulation of a working hypothesis or diagnosis about the current situation	“Patient is becoming more tachycardic, do you think there is a lot of bleeding?” (Trainee; Video MH 3–10)
	Recapping	Various pieces of information are brought together and a summary is provided	“The patient is in mid thirties. She's scheduled for lap chole. She started to develop tachycardia and hyperthermia at an undisclosed rate shortly we have given an antibiotic and contacted MH hotline.” (Trainee; Video MH 7–10)
	Explicitly assessing the situation	Providing an explicit judgment for something, give value to a certain process, information or strategy; this can be the process of evaluation information that has been gained through seeking information	“Paralysis: Patient seems “tight”, make sure she is paralyzed. (Trainee; Video MH 5–4)
	Reasoning	Explaining why something is more important than something else or why a specific behavior needs to be done	“Elevated CO2: this could be due to difficulty ventilating or drug reaction” (Trainee; Video MH 11–4)
Planning	Stating plans and priorities	Laying out the course of action for the next few minutes. Needs to contain at least 2 actions to show a sequence of actions	“Going to stop the agent and go up on flows” (Trainee; Video MH 6–11)
Implementation		States they are doing the task or delegates a task to another team member	“Administer dantrolene now” (Trainee; Video MH 5–11)

Two researchers coded six out of 22 randomly selected data files. The researchers discussed findings and resolved discrepancies through the process of social moderation. Cohen’s kappa interrater reliability was 0.73 [30]. The two researchers then independently annotated the remaining dataset.

#### Step 3: ordered network analysis

We applied ONA to our annotated data using the ordered network analysis R package [31, 32]. To conduct ONA, several parameters need to be specified, including: units of analysis, conversations, moving stanza window size, and codes. We defined the units of analysis as all lines of data associated with TuRBO annotated sentences subsetted by the higher- and lower-performing team identifiers. Since the naturally occurring interactions between anesthesia team members are done as exchanges of short phrases and sentences in video episodes, we defined video episodes as conversations. Within each conversation, the ONA algorithm uses a moving stanza window to slide through the conversation and record how codes in the current line are connected to codes that occur previously within the recent temporal context [33]. In this study, a moving stanza window of 4 lines (each line plus the 3 previous lines) was applied since the team members took on average 4 sentences to exchange information on the same topic. To compare communication patterns between higher- and lower-performing teams, we constructed ONA network visualizations for each group and analyzed two key parameters: (1) the frequency and types of TuRBO codes used, and (2) the temporal relationships between these codes. The ONA algorithm generated a multidimensional space where each team’s communication pattern was represented as a point, with the distance between points indicating similarity in communication patterns. We then conducted a two-sample t-test (assuming unequal variance) comparing the mean point positions of high- and low-performing teams in this projected ONA space. This analysis tested whether the overall communication patterns, including both code frequencies and their temporal sequences, differed significantly between high- and low-performing teams.

Networks were visualized using network graphs where nodes correspond to the codes, and lines connecting the nodes reflect the relative frequency of co-occurrence, or connection, between two codes. Node size indicates frequency of occurrence of the code and thickness of edges shows the strength of the relationship. The ONA model normalized the networks for all units of analysis before they were subjected to a dimensional reduction, which accounts for the fact that different units of analysis may have different amounts of coded lines in the data. For the dimensional reduction, we used a singular value decomposition, which produces orthogonal dimensions that maximize the variance explained by each dimension.

## Results

Five of the 22 (23%) anesthesia teams were determined to be high-performing based on administering dantrolene at < 10 min and/or performing other actions consistent with appropriate treatment such as calling the MH hotline and requesting a cognitive aid (as opposed to having to be prompted to perform these actions by a confederate in the scenario). All teams eventually administered dantrolene. The ranges and average times for completing the identified communication statements are included in Table 2.

**Table 2:** Performance Timing Analysis: Critical Response Metrics in Simulated MH Crisis Management Between High (*n* = 5) and Low (*n* = 17) Performing Teams

	Time to call for help (Range/Mean in minutes:seconds)	Time to ask surgeon to pause (Range/Mean in minutes:seconds)	Time to administer dantrolene (Range/Mean in minutes:seconds)	Required prompt from surgeon (N/%)
Low-performing teams (*N* = 17)	*N* = 17 2:22–7:24/5:57	*N* = 8 4:53–11:59/8:10	*N* = 17 9:57–17:47/12:41	1 (6%)
High-performing teams (*N* = 5)	*N* = 5 4:35—6:38/5:42	*N* = 5 6:07–8:55/7:17	*N* = 5 7:23–12:20/9:35	0 (0%)

**Fig. 2:**
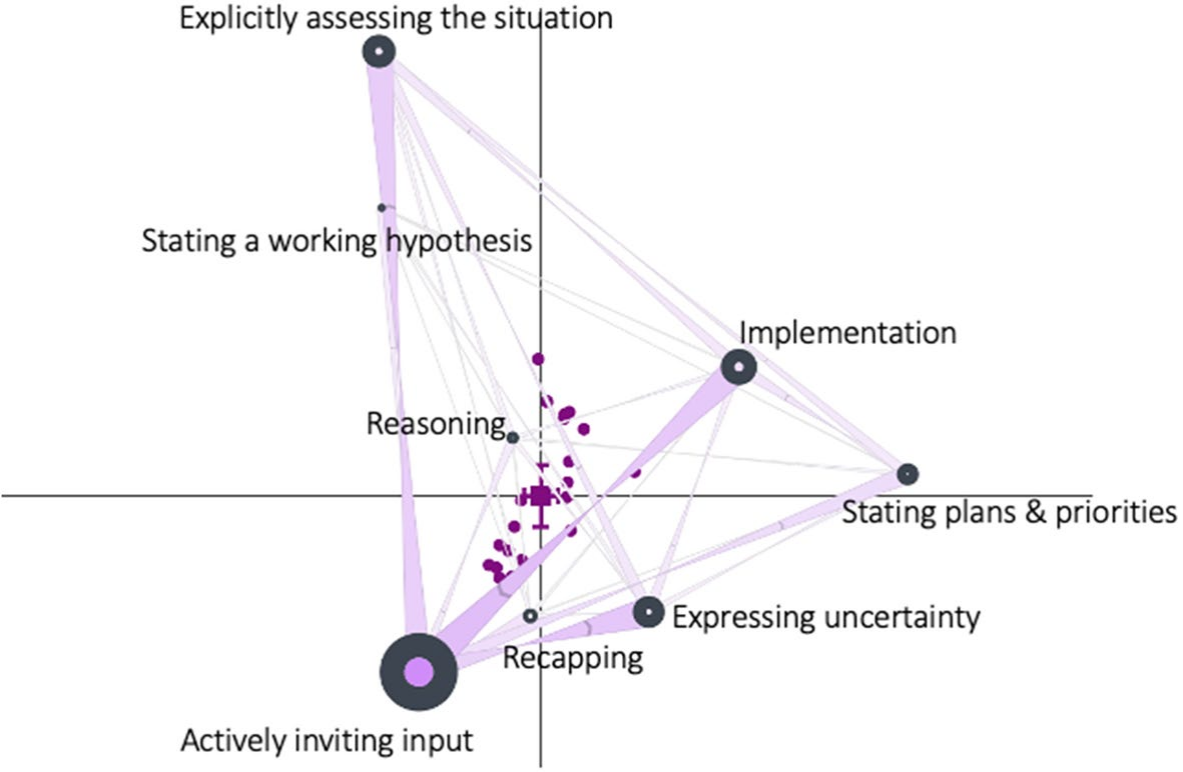
ONA mean network summarizing all 22 teams’ interactions. *Note: purple circles are plotted points for the individual team models, and black nodes represent the codes, the colored circle within a node represents self-connections. The larger the node size is, the higher frequency the code is being used as a response to other nodes. Directed connections are represented as triangles, with thicker and more saturated triangles represent stronger connections. The chevrons on the triangles indicate the direction of connections

Data from all 22 simulations were compiled into a single network analysis for an overall comparison of associations (Fig. 2), which demonstrated the strongest associations between Actively inviting input, Explicitly assessing the situation, Expressing uncertainty, and Implementation. Modest associations were most commonly seen involving Reasoning, Recapping, and Stating a working hypothesis.

**Fig. 3:**
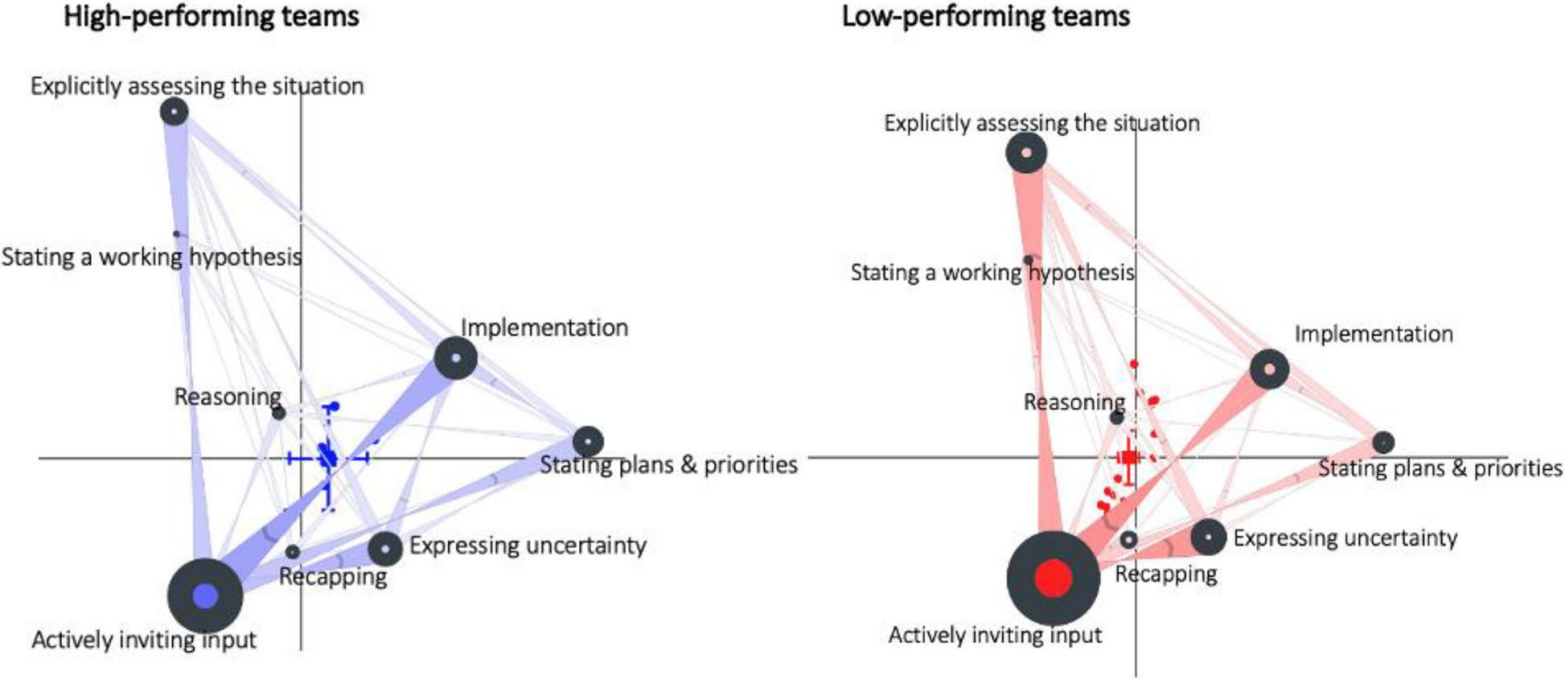
Mean ordered networks for the high-performing teams (in blue; left) and low-performing teams (in red; right)

Figure 3 shows the mean plotted point position for ONA networks for high- vs. low performing teams. Figure 3 shows both the frequency and order of TuRBO codes. A two sample t test showed significantly different communication patterns between high performing teams (mean = 0.18, SD = 0.19, *N* = 5) and low performing teams (mean = −0.05, SD = 0.12, *N* = 17; t(5.01) = −2.65, *p* = 0.04, Cohen’s d = 1.72; at the alpha = 0.05 level). High-performing teams engaged in more action-oriented communication patterns, characterized by higher frequencies of Implementation and Stating plans and priorities codes, while low-performing teams tend to remain in information-gathering communication patterns, characterized by frequently repeated cycles of Actively inviting input and Explicitly assessing the situation codes (see Fig. 3).

The strongest associations were also seen between Actively inviting input and Implementation as well as Explicitly assessing the situation and Actively inviting input for high-performing teams. Low performing teams frequently cycled between Explicitly assessing the situation and Actively inviting input as well but with fewer transitions to Implementation behaviors in comparison to high performing teams. Below are two examples to illustrate these patterns:

*Trainee:* “We're actually becoming tachycardic, the patient's temperature has gone up a little bit, end-tidal CO2 are a little high.” [Explicitly assessing the situation]

*Trainee:* “I'm gonna try to breathe her down, but we might have a reaction, sir.” [Stating plans and priorities]

*Surgeon:* “Ok… She was septic enough from the beginning” [Reasoning]

*Trainee:* “Hey, Don [nurse’s name]. You know what's going on. I got tachycardia. Actually the temperature is going up real fast. You know what, would you go get the MH kit for us? We might have to start some dantrolene [Implementation]

In this example, the high performing team quickly assessed the situation, stated a plan, provided reasoning, and assigned a task to address the likely malignant hyperthermia. The trainee rapidly diagnosed the problem and initiated appropriate action rather than getting stuck exploring hypotheses.

*Trainee:* “So what's happening back here: she's not stable right now so her CO2 is rising and peak pressures are up.” [Actively inviting input]

*Surgeon:* “Do you think its my insufflation doing that? [Stating a working hypothesis]

*Trainee:* “Possibly. Can you take down your insufflation and let's see where we're at?” [Expressing uncertainty]

*Trainee:* “CO2 is elevated and her peak pressures are high.” [Explicitly assessing the situation]

*Trainee:* “You got any thoughts?” [Actively inviting input]

*Support team member:* “She having a PE potentially?” [Stating a working hypothesis]

*Trainee:* “Maybe. That's her temperature [points at the monitor], but we got peak pressures problems. The peak pressures and her heart rate and this is the best pressure I can get. I've given 400 of neosynephrine.” [Recapping]

*Surgeon:* “I think we should do something about that. She's still tight and I want to get done here.” [Stating plans and priorities]

*Trainee:* “Okay. I understand. Think it might be PE. She's a smoker and she's on oral contraceptive. Did she get anything lovenox? Has she been on any blood thinners at all?” [Stating a working hypothesis] [Actively inviting input]

In this example, the trainee engaged in circular discussion, inviting input and stating hypotheses without moving to implementation. The trainee spun in exploration while the surgeon applied pressure to complete the operation.

## Discussion

This study highlights the potential of measuring and modeling team behaviors using network analysis as a promising technique for exploring patterns of learner success and decision-making patterns within simulated environments. While this approach offers valuable insights into how teams might function, identified patterns that resulted from this analysis cannot be generalized to similar real-world clinical situations without extensive additional analysis of the fidelity of the simulation scenario. The simulation scenario used in our study may have influenced the interaction dynamics to focus more on evaluating individual anesthesiologist performance than on measuring the collaborative problem-solving that typically occurs in a real operating room. While this study design allowed for standardized examination of individual crisis management skills, it limits the generalizability of our findings to more authentic team dynamics. However, the methodological approach demonstrated here for analyzing and modeling team behaviors can be of value for simulation educators and researchers seeking to enhance training methods.

For simulation educators, this study provides a methodological blueprint that can inform debriefing strategies. Educators can leverage TuRBO or similar rubrics [34, 35] to systematically gather and analyze team interaction data using ONA, thereby enhancing the quality and specificity of feedback provided during training sessions [36, 37]. For example, understanding communication bottlenecks and streamlining transitions from information gathering to implementation can inform improvement targets during debrief sessions. In this study, the rich network visualizations showed exactly how and when decision making and communications falter, and what clinical management and non-technical practices should be improved upon, reinforced, or avoided at best. Our analysis spotlighted key similarities and differences between high and low performing teams in terms of their communication patterns and decision-making. This is helpful for debriefing of performance and providing feedback as well as developing curriculum and for training and practice. For instance, in our analysis, the least frequent categories are: “stating hypothesis”, “reasoning”, and “re-capping”. These insights would highlight the team’s need to focus on summarizing behavior and could be incorporated as a teaching point [38]. Summarizing and recapping frequently during critical care can promote a shared mental model. In addition, encouraging team members to speak up, offer input and share one's thoughts/observations during such recapping periods can further promote shared understanding in a team [39].

Similarly, educators can use network visualizations to demonstrate how low-performing teams often get caught in cycles of information gathering and planning without progressing to implementation, as shown in Fig. 3. To address this, educators could develop targeted exercises that help teams recognize these communication loops and practice transitioning more efficiently from assessment to action. Specific training interventions might include: (1) incorporating deliberate pauses during simulations to help trainees identify when and why they are stuck in assessment cycles, and (2) using video playback with overlaid network analysis to show teams their communication patterns. Additionally, educators can use exemplar cases from high-performing teams to demonstrate effective transitions from information gathering to implementation, highlighting how efficient decision-making and clear task delegation contribute to better outcomes.

For researchers, this study demonstrates both the promise and challenges of analyzing dynamic team processes and performance in healthcare settings. The combination of ONA and similar coding schemes such as TuRBO offers a methodological framework that could be extended to study authentic clinical teams in real-world settings (see notable example [19]). Researchers could explore integrating automated speech recognition and natural language processing technologies [40, 41], including large language models, to enable real-time analysis of team communications, while being mindful of technical challenges such as speaker diarization and crosstalk in clinical environments. Standardizing data collection and storage protocols through established APIs would facilitate data sharing across institutions and enable larger-scale studies of team dynamics. Future research should also focus on developing more granular behavioral codes that can better capture multimodal (e.g., visual attention, cognitive load, linguistic and acoustic speech patterns) aspects of team processes. Multimodal data may afford researchers and educators to identify subtle markers that could predispose trainees to errors or delays in therapeutic interventions. By integrating these diverse data streams, we can develop machine learning algorithms that reliably predict and provide “just in time, just for you, and just enough” support to individual learners and teams. Additionally, researchers should investigate how communication patterns might differ between simulated and real clinical environments, and how these differences might impact team measures and training across different clinical scenarios, team compositions, and healthcare settings. Finally, researchers should explore how these tools could support both real-time feedback during actual clinical care and post-event debriefing [42], while carefully evaluating the impact of such tools on individual and team learning gains, quality of debriefing, and ultimately patient outcomes.

## Conclusion

This study demonstrated a novel combination of video analysis, behavioral coding, and network analysis to quantify and compare communication patterns in high- versus low-performing care teams. Importantly, traditional multivariate techniques face two major challenges when analyzing complex interactional data with many variables: scalability and interpretability. As the number of variables increases, the potential interactions between them grow exponentially, requiring extremely large datasets even for models with a moderate number of interacting elements. Additionally, most traditional multivariate techniques do not produce visualizations that allow for easy interpretation of the underlying model and interaction patterns. The approach demonstrated here addresses these challenges by enabling a more robust measurement of specific teamwork patterns that enable or impede crisis management skills. This approach aligns with calls to utilize network science in studying healthcare teams [43, 44]. By revealing optimal and suboptimal communication patterns, ONA provides an evidence base for improving communication between physicians and nurses. By mapping these contrasting communication workflows, this study showcases how ONA can pinpoint bottlenecks and vulnerabilities in team coordination during acute care scenarios. Such contextualized insights can guide the development of targeted educational interventions and feedback to optimize communication practices aligned with expert performance.

## Data Availability

The datasets used and/or analysed during the current study are available from the corresponding author on reasonable request.

## Abbreviations

ONA: Ordered network analysis
MH: Malignant hyperthermia
TuRBO: Team Reflection Behavioral Observation framework
MOCA: Maintenance of Certification in Anesthesiology
ASA: American Society of Anesthesiologists
ABA: American Board of Anesthesiologists
